# Biocidal Agents Used for Disinfection Can Enhance Antibiotic Resistance in Gram-Negative Species

**DOI:** 10.3390/antibiotics7040110

**Published:** 2018-12-14

**Authors:** Günter Kampf

**Affiliations:** University Medicine Greifswald, Institute for Hygiene and Environmental Medicine, 17475 Greifswald, Germany; guenter.kampf@uni-greifswald.de

**Keywords:** biocide, cross-resistance, cross-tolerance, antibiotics, antiseptic stewardship

## Abstract

Biocidal agents used for disinfection are usually not suspected to enhance cross-resistance to antibiotics. The aim of this review was therefore to evaluate the effect of 13 biocidal agents at sublethal concentrations on antibiotic resistance in Gram-negative species. A medline search was performed for each biocidal agent on antibiotic tolerance, antibiotic resistance, horizontal gene transfer, and efflux pump. In cells adapted to benzalkonium chloride a new resistance was most frequently found to ampicillin (eight species), cefotaxime (six species), and sulfamethoxazole (three species), some of them with relevance for healthcare-associated infections such as *Enterobacter cloacae* or *Escherichia coli*. With chlorhexidine a new resistance was often found to ceftazidime, sulfamethoxazole and imipenem (eight species each) as well as cefotaxime and tetracycline (seven species each). Cross-resistance to antibiotics was also found with triclosan, octenidine, sodium hypochlorite, and didecyldimethylammonium chloride. No cross-resistance to antibiotics has been described after low level exposure to ethanol, propanol, peracetic acid, polyhexanide, povidone iodine, glutaraldehyde, and hydrogen peroxide. Taking into account that some biocidal agents used in disinfectants have no health benefit (e.g., in alcohol-based hand rubs) but may cause antibiotic resistance it is obvious to prefer products without them.

## 1. Introduction

Biocidal agents used for disinfection are one of many elements to limit the spread of antibiotic resistant bacteria. Most users of disinfectants would not expect that biocidal agents may cause antibiotic resistance themselves. Triclosan is such an example. It was used for decades in antimicrobial soaps in the US and considered to be safe and effective [1]. But in 2016 19 active ingredients including triclosan were banned by the US Food and Drug Administration for antimicrobial soaps used at home by the general population [2]. The decision was justified by associated risks including antibiotic resistance and a lack of a health benefit: “A risk must be balanced that demonstrate a direct clinical benefit (i.e., a reduction of infection)—that the product is superior to washing with non-antibacterial soap and water in reducing infection.” The scientific community welcomed the decision: “We applaud this rule specifically because of the associated risks that triclosan poses to the spread of antibiotic resistance throughout the environment. This persistent chemical constantly stresses bacteria to adapt, and behavior that promotes antibiotic resistance needs to be stopped immediately when the benefits are null” [3].

Other biocidal agents used for disinfection in healthcare, veterinary medicine, food production, food handling or in the domestic setting may also have a risk of enhancing antibiotic resistance, especially during low level exposure [4]. Persistence of the biocidal agent is certainly an advantage for an adaptive response. However, there is currently a lack of awareness in the infection control community that some biocidal agents may have a larger risk for promoting antibiotic resistance than others. The aim of the review is therefore to summarize data on the development of antibiotic tolerance and resistance, changes of horizontal gene transfer, induction of antibiotic resistance genes, and the effect on common efflux pump genes in Gram-negative species caused by low level exposure to commonly used biocidal agents.

The following biocidal agents were reviewed: triclosan, benzalkonium chloride, hydrogen peroxide, glutaraldehyde, ethanol, chlorhexidine digluconate, sodium hypochlorite, didecyldimethylammonium chloride (DDAC), octenidine, peracetic acid, propanol, polihexanide, and povidone iodine. The medline search for “horizontal gene transfer”, antibiotic, and each biocidal agent on 31 August 2018 revealed four hits for hydrogen peroxide, three hits for ethanol, three hits for chlorhexidine digluconate, two hits for triclosan, one hit for benzalkonium chloride, and 0 hits for glutaraldehyde, sodium hypochlorite, propanol, povidone iodine, peracetic acid, polihexanide, DDAC and octenidine. The medline search for “cross tolerance”, antibiotic, and each biocidal agent on 19 November 2018 revealed five hits for benzalkonium chloride and hydrogen peroxide, three hits for ethanol, two hits for sodium hypochlorite and povidone iodine, one hit for chlorhexidine digluconate and polihexanide, and 0 hits for propanol, DDAC, octenidine, and peracetic acid. The medline search for “cross resistance”, antibiotic and each biocidal agent on 19 November 2018 revealed 44 hits for triclosan, 36 hits for benzalkonium chloride, 32 hits for hydrogen peroxide, 23 hits for ethanol, 22 hits for povidone iodine, 19 hits for glutaraldehyde, 11 hits for sodium hypochlorite, eight hits for chlorhexidine digluconate, seven hits for peracetic acid, five hits for triclosan, two hits for propanol, polihexanide, octenidine, and glutaraldehyde, and one hit for DDAC. The medline search for “efflux pump”, antibiotic and each biocidal agent on 19 November 2018 revealed 35 hits for triclosan, 31 hits for benzalkonium chloride, 10 hits for hydrogen peroxide, three hits for glutaraldehyde and ethanol, two hits for chlorhexidine digluconate, one hit for sodium hypochlorite and 0 hits for DDAC, octenidine, peracetic acid, propanol, polihexanide, and povidone iodine.

Publications were included and results were extracted from them when they provided original data on an adaptive response to the exposure of Gram-negative bacteria to sublethal concentrations of the biocidal agents described above resulting in a tolerance or resistance to antibiotics including antibiotic resistance gene changes, in a change of efflux pump activity or horizontal gene transfer. Articles were excluded when they described changes in Gram-positive species, fungi or mycobacteria. Reviews were also excluded but screened for any information within the scope of the review.

## 2. Benzalkonium Chloride

### 2.1. Antibiotic Tolerance or Resistance after Low Level Biocide Exposure

In one study it was described that exposure of *Escherichia coli* to low level benzalkonium chloride can increase the tolerance to benzalkonium chloride 2.6-fold and in addition also 3.3-fold to 7-fold to various antibiotics. A classification to susceptibility categories, however, was not found (Table 1).

In 11 studies an associated increase of tolerance or a new resistance to antibiotics was described for some Gram-negative species (Table 2). A new resistance was most frequently found to ampicillin (eight species), cefotaxime (six species), and sulfamethoxazole (three species). For two species a new antibiotic resistance was detected for ceftazidime, trimethoprim-sulfamethoxazol, trimethoprim, tetracycline, imipenem, chloramphenicol, amoxicillin, or amoxicillin-clavulanic acid. Only one species was resistant to nalidixic acid or ceftriaxone. Among the species some have a major relevance for healthcare-associated infections such as *Enterobacter cloacae* or *Escherichia coli*.

### 2.2. Effect on Antibiotic Resistance Genes

Benzalkonium chloride has been described to co-select for other antimicrobial resistance genes [17].

### 2.3. Increase of Horizontal Gene Transfer

The general possibility of horizontal gene transfer for the spread of antibiotic and biocide resistance has been described already in 2001 [18]. In two of 179 *Escherichia coli* isolates from retail food qacH-associated integrons associated with tolerance to benzalkonium chloride located on 100 kb plasmids could be transferred to an *E. coli* recipient, indicating the co-existence and co-dissemination of disinfectant and antimicrobial resistance genes among bacterial species [19].

### 2.4. Induction of Common Efflux Pumps

In *Pseudomonas aeruginosa*, benzalkonium chloride can induce the MexCD-OprJ multidrug efflux pump [20].

### 2.5. Additional Findings

Some other studies demonstrate a correlation between tolerance to benzalkonium chloride and resistance to various antibiotics. In 153 *Escherichia coli* blood culture isolates, for example, a higher MIC of benzalkonium chloride was associated with a decreased susceptibility to cotrimoxazole [21]. In 52 *Pseudomonas* spp. from meat chain production, a correlation between resistance to benzalkonium chloride and ampicillin, amoxicillin, erythromycin, and trimethoprim was found [22]. Repeated in vitro exposure of *Salmonella Typhimurium* cells to quaternary ammonium compounds selects for a higher tolerance to chloramphenicol, tetracycline, ampicillin, and acriflavine which is explained by an overexpression of the AcrAB efflux pump [23]. Few other studies do not describe such a correlation. No correlation between multiple antibiotic-resistant bacteria and a tolerance to benzalkonium chloride was found in 122 isolates of *Salmonella* spp. from poultry and swine [24]. In analogy, no association between resistance to multiple antibiotic and quaternary ammonium compounds was found in 103 Gram-negative clinical isolates [25]. One of the reasons for a cross-resistance with benzalkonium chloride is a multidrug efflux protein MdtM which was detected in *E. coli*. It belongs to the large and ubiquitous major facilitator superfamily (MFS). Benzalkonium chloride, didecyldimethylammonium chloride and some antibiotics are among the substrates transported by MdtM [26]. It was also shown with *E. coli* that many redundant multidrug resistance transporters also enhance biofilm formation and drug tolerance including benzalkonium chloride [27]. Efflux pumps also explain resistance to benzalkonium chloride in *P. aeruginosa* [12]. In *P. fluorescens* high level resistance to benzalkonium chloride was also explained by an efflux system which excretes only specific cationic disinfectants belonging to the group of quaternary ammonium compounds [28]. Over-expression of efflux pumps AcrAB or AcrEF was detected in benzalkonium chloride-resistant mutants of *S. Typhimurium* [29].

## 3. Chlorhexidine Digluconate

### 3.1. Antibiotic Tolerance or Resistance after Low Level Biocide Exposure

Six studies indicate that low level chlorhexidine exposure quite often results in an antibiotic resistance, so far mainly described in biocide-sensitive strains from organic foods (Table 3). A new resistance was most frequently found to ceftazidime, sulfamethoxazole, and imipenem (eight species each) as well as cefotaxime and tetracycline (seven species each). For two species a new antibiotic resistance was detected for ampicillin. Only one species was finally resistant to nalidixic acid, colistin, or tobramycin. Among the species some have also relevance for healthcare such as *Enterobacter cloacae*, *Escherichia coli*, or *Klebsiella pneumoniae*.

### 3.2. Increase of Horizontal Gene Transfer

Horizontal transfer of mobile antibiotic resistance elements by conjugation could be significantly increased by low level exposure to chlorhexidine digluconate (24.4 µg/L) to a recipient *Escherichia coli* strain [34]. In addition, an additional sh-fabI allele was detected in clinical isolates of *Staphylococcus aureus* derived from *Staphylococcus haemolyticus* suggesting a high potential of its horizontal gene transfer [35].

### 3.3. Induction of Common Efflux Pumps

Chlorhexidine was able to induce the expression of 6 efflux pump genes (bmeB1, bmeB3, bmeB4, bmeB7, bfrA1 and bfrA2) in *Bacteroides fragilis* ATCC 25285 exposed for 12 h to 0.06% chlorhexidine [30]. It can also induce the MexCD-OprJ multidrug efflux pump in *Pseudomonas aeruginosa* [20,36].

### 3.4. Additional Findings

A similar overall result was found for low level chlorhexidine exposure. Some additional studies demonstrate a correlation between tolerance to chlorhexidine and resistance to various antibiotics. A positive correlation between resistance to some biocidal agents (cetrimide, chlorhexidine, hexachlorophene) and to antibiotics was described in 1991 for *Serratia marcescens* and *Alcaligenes* spp. [37]. In 49 *Acinetobacter baumannii* strains with a reduced susceptibility to chlorhexidine a co-resistance to carbapenem, aminoglycoside, tetracyclin, and ciprofloxacin was found [38]. In *Bacteroides fragilis* multiple antibiotic resistance was induced by a 2.7–6.0-fold increase of 6 efflux pumps [30]. In an *Escherichia coli* strain an unstable resistance to tobramycin was detected after low level exposure to chlorhexidine for up to 24 h [32]. In Trinidad 11 of 120 chlorhexidine solutions were found to be contaminated with *Pseudomonas* spp., with resistance rates to ciprofloxacin of 58.3%, to norfloxacin of 50.0%, to tobramycin of 45.8%, and to gentamicin with 41.7% [39]. In a chlorhexidine-resistant *Pseudomonas stutzeri* isolate a cross-resistance to polymyxin and gentamicin was found [40]. A study with six other *Pseudomonas stutzeri* strains revealed a cross-resistance to ampicillin in five strains, to polymyxin in four strains, to erythromycin in three strains, and to nalidixic acid and gentamicin in two strains after low level exposure to chlorhexidine diacetate for six weeks [41]. Some authors found no cross-resistance between chlorhexidine and antibiotics. For example, no correlation was found between the susceptibility to chlorhexidine and 10 different antibiotics among 101 genetically distinct isolates of the *B. cepacia* complex [42]. No cross-resistance was found between chlorhexidine and five antibiotics in 130 *Salmonella* spp. from two turkey farms [43]. And no correlation between resistance to chlorhexidine and 16 different antibiotics was found in 52 *Pseudomonas* spp. from meat chain production [22]. A possible cross-resistance between chlorhexidine and antibiotics is discussed controversially [44,45]. As an example, the widespread use of chlorhexidine has not yet resulted in a clinically relevant resistance to antibiotics [46,47] even though the development of resistance to these agents is regarded as realistic [48].

## 4. Triclosan

### 4.1. Antibiotic Tolerance or Resistance after Low Level Biocide Exposure

Five studies indicate that low-level triclosan exposure may cause antibiotic resistance, so far also mainly described in biocide-sensitive strains from organic foods (Table 4). A new resistance was most frequently found to sulfamethoxazole (five species), ampicillin or cefotaxime (four species each) and ceftazidime, trimethoprim or chloramphenicol (three species). For two species a new antibiotic resistance was detected for amoxicillin-clavulanic acid, trimethoprim-sulfamethoxazol, amoxicillin, nalidixic acid, tetracycline, or imipenem. Only one species was resistant to erythromycin, ceftiofur, or cefoxitin. One of the species has a major relevance for healthcare-associated infections (*Escherichia coli*). The effect in *Escherichia coli* is partly explained by changes in bacterial membrane properties and enhancing the efflux system [49].

### 4.2. Increase of Horizontal Gene Transfer

Horizontal transfer of mobile antibiotic resistance elements by conjugation could be significantly increased by low level exposure to triclosan (0.1 mg/L) to a recipient *Escherichia coli* strain [34].

### 4.3. Additional Findings

Triclosan is also a biocidal agent which can enhance resistance to antibiotics in some Gram-negative species. An associated cross-tolerance or cross-resistance between triclosan and various antibiotics seems uncommon in *Acinetobacter johnsonii* and *Escherichia coli* [53] although one study has described a cross-tolerance between triclosan and chloramphenicol (intermediate susceptibility) in an *Acinetobacter johnsonii* strain [54]. Among 52 *Pseudomonas* spp. isolates from meat chain production, a general cross-tolerance between triclosan and ampicillin, amoxicillin, erythromycin, imipenem and trimethoprim was described [22]. Resistance in *Salmonella* caused by increasing concentrations of triclosan is associated with an overexpression of the AcrAB efflux pump [23]. A possible mechanism was shown with *Agrobacterium tumefaciens* where triclosan abolishes the interaction between the transcriptional repressor of the acrABR operon (acrR) and the DNA to which acrR specifically binds in the acrA promoter region [55]. A correlation between a decreased triclosan susceptibility and multidrug-resistance was shown in 428 *Salmonella enterica* isolates. Four percent of the isolates were triclosan-tolerant, 56% of them were multidrug-resistant. Among the remaining triclosan-sensitive isolates only 12% were multidrug-resistant [56]. Efflux pumps were also considered to explain a lower susceptibility to triclosan in antibiotic-resistant *Escherichia coli* and *Salmonella* spp. isolated from poultry and clinical samples [57]. In the domestic setting no antibiotic and antibacterial agent cross-resistance in target bacteria from antibacterial product users and nonusers was found [58].

## 5. Didecyldimethylammonium Chloride

### 5.1. Antibiotic Tolerance or Resistance after Low Level Biocide Exposure

One study was found with data for 2 species (Table 5). 59% of 54 *Escherichia coli* strains became multiresistant to antibiotics after low level didecyldimethylammonium chloride exposure whereas a new resistance to at least one antibiotic occurred in only 13% of 54 *Salmonella enterica* strains.

### 5.2. Additional Findings

Fewer data are available with didecyldimethylammonium chloride. Some studies describe a cross-tolerance between didecyldimethylammonium chloride and antibiotics. For example, in 153 *E. coli* blood culture isolates a higher MIC of didecyldimethylammonium chloride was associated with a decreased susceptibility to cotrimoxazole [21]. In *E. coli* didecyldimethylammonium chloride-MICs were positively correlated with MICs of piperacillin and sulphamethoxazole-trimethoprim [60]. However exposure of *A. baumannii*, *C. sakazakii*, *E. coli*, *P. aeruginosa* and *P. putida* to increasing didecyldimethylammonium chloride concentrations over 14 passages of four days each did not result in antibiotic resistance [61].

## 6. Sodium Hypochlorite

### 6.1. Antibiotic Tolerance or Resistance after Low Level Biocide Exposure

Some strains of *Salmonella* species, adapted to sodium hypochlorite, have occasionally developed an associated resistance to specific antibiotics such as gentamicin in *S. Anatum*, ceftazidime in *S. Enteritidis*, amikacin, ampicillin, chloramphenicol and nitrafurantoin in *S. Hadar*, gentamicin, ceftazidime, amikacin, tobramycin, cefoxitin, and tetracycline in *S. Infantis*, amikacin and ampicillin/sulbactam in *S. Kentucky*, gentamicin, ceftazidime, tobramycin, cefoxitin, cefazolin and nalidixic acid in *S. Thompson*, amikacin, tobramycin, cefazolin, cefotaxime in *S. Thyphimurium*, teicoplanin in *S. Virchow*, and gentamicin, nitrafurantoin, cephalothin, cefepime and enrofloxacin in *Salmonella* spp. strain 1,4, [5],12:i- [62]. It is particularly interesting that an *E. coli* strain was found to be viable but non-culturable after low level exposure to sodium hypochlorite and that the same adapted cells were able to better persist in the presence of various antibiotics [63].

### 6.2. Effect on Antibiotic Resistance Genes

Sodium hypochlorite can reduce antibiotic resistance genes or plasmids to some extent (mostly ≤ 1.0 log). This effect has been shown with three antibiotic resistance genes (sul1, blaTEM, blaCTX-M) which were reduced by 0.8–0.9 log. The antibiotic resistance plasmid pB10 from an *E. coli* strain was also reduced by 1.0 log [64]. A somatic coliphage could be reduced in 30 min by at least 1.0 log. The antibiotic resistance genes, however, were not significantly reduced (0.2–0.6 log) [65]. Similar findings were reported with the tet(W) gene in *Acinetobacter*, *Aeromonas*, *Chryseobacterium*, *E. coli*, *Pseudomonas* and *Serratia*. It was mostly reduced by 0.0–0.9 log immediately after exposure to sodium hypochlorite, the effect was stronger in *Acinetobacter* (1.8 log) and *Chryseobacterium* (4.0 log) [66]. A higher concentration of active chlorine (range: 2–32 mg/L) decreases the abundance of antibiotic resistance genes in wastewater linearly [67]. Bacteria may, however, persist after sodium hypochlorite treatment. Survivors may outgrow from the biofilm which may increase the level of antibiotic resistance genes in water. Sodium hypochlorite at 1 mg/L can destroy the piperazine ring of ciprofloxacin in drinking water distribution systems. As a consequence, specific antibiotic resistance genes increased in effluents (e.g., mexA and qnrS) and others increased in biofilms (qnrA and qnrB). These bacterial genera seem to grow by transformation of ciprofloxacin chlorination products in drinking water distribution systems [68].

## 7. Other Biocidal Agents

### 7.1. Antibiotic Tolerance or Resistance after Low Level Biocide Exposure

Cross-tolerance between octenidine and gentamicin, colistin, amikacin, and tobramycin has been described in a *Pseudomonas aeruginosa* isolate [69]. No cross-tolerance or cross-resistance to antibiotics has so far been described after low level exposure to ethanol, propanol, peracetic acid, polyhexanide, povidone iodine, glutaraldehyde, and hydrogen peroxide.

### 7.2. Effect on Antibiotic Resistance Genes

The data for peracetic acid are not so clear [70]. Peracetic acid in waste water was shown to stimulate the selection of antibiotic resistance genes [71]. It was, however, not able to reduce nine antibiotic resistance genes (ampC, mecA, ermB, sul1, sul2, tetA, tetO, tetW, vanA) in wastewater [72]. For triclosan, didecyldimethylammonium chloride, povidone iodine, octenidine, polyhexanide, glutaraldehyde, hydrogen peroxide, ethanol, and propanol no data on a possible induction of antibiotic resistance genes or a reduction of antibiotic resistance genes were found.

### 7.3. Increase of Horizontal Gene Transfer

Production of hydrogen peroxide in cells of *Streptococcus gordonii* was shown to cause release of extracellular DNA which may serve as a pool for novel genetic traits such as antimicrobial resistance [73]. Hydrogen peroxide produced by one species is able to induce the DNA release by another which has important implications for the role of hydrogen peroxide in interspecies horizontal gene transfer [73]. Whether this finding has any relevance for extracellular low-level hydrogen peroxide exposure is unknown. Low level chlorination of 0.3–0.5 mg/L chlorine was able to decrease conjugative transfer of the RP4 plasmid in drinking water [74]. No effect on horizontal gene transfer by low-level exposure was so far described for ethanol, propanol, peracetic acid, glutaraldehyde, polihexanide, DDAC, octenidine, and povidone iodine.

### 7.4. Additional Findings

Hydrogen peroxide and peracetic acid were not among the biocidal agents with evidence that low level exposure can cause antibiotic resistance. This is probably explained by their lower stability which may make it more difficult for bacteria to adapt to the biocidal agents. Another advantage for peracetic acid in this context is that it was able to transform different beta-lactam antibiotics in wastewater at concentrations of 0.0005–0.002% which may help to reduce antibiotic selection pressure in wastewater [75]. 

## 8. Discussion

The health burden of five types of infection with antibiotic-resistant bacteria is high in Europe with an estimated 671,689 infections in 2015, of which 63.5% were associated with healthcare [76]. Antibiotic resistance caused by some biocidal agents is very likely of minor relevance in this context. But nevertheless it seems necessary to critically review disinfectant formulations with the aim to ban any unnecessary selection pressure.

One example is alcohol-based hand rubs. Some products contain in addition to the alcohol(s) non-volatile biocidal agents such as chlorhexidine digluconate, triclosan, benzalkonium chloride, didecyldimethylammonium chloride, polihexanide, or octenidine dihydrochloride [77]. A recent review with some of the agents shows that all formulations containing such an additional biocidal agent fail to show a superior bactericidal efficacy according to EN 12791 after three hours under the surgical glove [78]. In addition, a health benefit (e.g., reduction of surgical site infection) has so far not been shown for any of the additional biocidal agents in alcohol-based hand rubs [79]. Taking into account that there is no health benefit for any of these additional biocidal agents for the application hand disinfection but a realistic potential to enhance the development of antibiotic resistance it seems logical and responsible to prefer alcohol-based hand rubs without additional biocidal agents as long as they have an equivalent user acceptability and efficacy for hand disinfection (“antiseptic stewardship”) [77]. The Commission for Hospital Hygiene and Infection Control (KRINKO) at the Robert Koch-Institute, Berlin, Germany, has therefore recommended that alcohol-based hand rubs with persistent biocidal agents cannot be recommended [80]. 

Additional biocidal agents in alcohol-based skin antiseptics should also be reviewed. Some products contain chlorhexidine, octenidine, povidone iodine, or benzalkonium chloride [81]. A proven health benefit (prevention of catheter-associated bloodstream infections and probably also surgical site infections) has so far only been shown for the additional chlorhexidine [82,83,84,85,86]. Additional octenidine may also have a health benefit for the prevention of catheter-associated bloodstream infections [87]. No health benefit has been shown for additional benzalkonium chloride or povidone iodine. For benzalkonium chloride at a low concentration there is even evidence that a persistent antimicrobial effect on the skin over 48 h is lacking [88]. The use of chlorhexidine in alcohol-based skin antiseptics seems reasonable despite some risks. The use of octenidine in alcohol-based skin antiseptics may also be favourable although the evidence for a health benefit is sparse. Benzalkonium chloride in alcohol-based skin antiseptics does not have any health benefit but has some relevant risks including antibiotic resistance development. It should be replaced [81].

For other types of applications such as surface disinfection, wound antisepsis, mucous membrane antisepsis, or instrument disinfection, preference should be given to those biocidal agents without or with a low selection pressure assuming that their antimicrobial activity, material compatibility, and user safety is at least as good for the intended use. Other antimicrobial agents such as cold plasma may be an alternative in the future [89].

## 9. Conclusions

Antibiotic resistance may occur after exposure of various Gram-negative species to sublethal concentrations of some biocidal agents such as benzalkonium chloride, chlorhexidine or triclosan. Their use as an antiseptic agent should be restricted to applications with a proven health benefit. General preference should be given to biocidal agents without or with a low selection pressure assuming that their antimicrobial activity, material compatibility, and user safety is at least as good for the intended use.

## Figures and Tables

**Table 1:** Gram-negative species with increased antibiotic tolerance after various types of low level exposure (<MIC value) to benzalkonium chloride (BAC).

Species	Strain(s)	MIC Increase (BAC)	Antibiotic(s)	MIC Increase (Antibiotic)	Reference
*Escherichia coli*	ATCC 25922 and 9 avian and porcine strains	2.6-fold	Florfenicol Cefotaxime Chloramphenicol Ceftazidime Nalidixic acid Ampicillin Tetracycline Ciprofloxacin Sulfamethoxazole Trimethoprim	7-fold ^1^ 6.3-fold ^1^ 6.1-fold ^1^ 4.8-fold ^1^ 4.4-fold ^1^ 4.3-fold ^1^ 4.2-fold ^1^ 3.8-fold ^1^ 3.7-fold ^1^ 3.3-fold ^1^	[5]

^1^ microdilution method (mg/L).

**Table 2:** Gram-negative species with antibiotic resistance after various types of low level exposure (<MIC value) to BAC.

Species	Strain(s)	MIC Increase (BAC)	Antibiotic(s)	Pre-Value	Post-Value	Category	Reference
*Burkholderia cepacia complex*	*B. lata* strain 383 (4 experiments)	-	Imipenem Meropenem Ciprofloxacin Ceftazidime Tobramycin	24 ^1^ 40.7 ^1^ 30 ^1^ 40.3 ^1^ 7.3 ^1^	16 (1) ^1^ 34–35.5 (2) ^1^ 12–24 (2) ^1^ 12 (1) ^1^ 0 (1) ^1^	- - - - -	[6]
*Chryseobacterium* spp.	Biocide-sensitive strain from organic foods	20-fold	Ampicillin	-	64^1^	R	[7]
*Enterobacter cloacae*	Two biocide-sensitive strains from organic foods	12-fold–30-fold	Cefotaxime Ampicillin	- -	128 (1) ^1^ 64 (1) ^1^	R R	[7]
*Enterobacter ludwigii*	Biocide-sensitive strain from organic foods	30-fold	Cefotaxime	-	128 ^1^	R	[7]
*Enterobacter* spp.	Six biocide-sensitive strains from organic foods	5-fold–300-fold	Ampicillin Sulfamethoxazol Ceftazidime Cefotaxime Trimethoprim-sulfamethoxazol	- - - - -	64 (5) ^1^ 1014 (2) ^1^ 64 (1) ^1^ 64 (1) ^1^ 8/152 (1) ^1^	R R R R R	[7]
*Escherichia coli*	ATCC 11775	6-fold	Ampicillin Chloramphenicol Erythromycin Gentamicin Kanamycin Nalidixic acid Norfloxacin Penicillin Tetracycline	10 ^1^ 10 ^1^ 140 ^1^ 2 ^1^ 8 ^1^ 8 ^1^ 0.15 ^1^ 250 ^1^ 4 ^1^	50 ^1^ 240 ^1^ 180 ^1^ 4 ^1^ 16 ^1^ 30 ^1^ 0.4 ^1^ 400 ^1^ 16 ^1^	- - - - - - - - -	[8]
*Escherichia coli*	DSM 682	6-fold	Ampicillin Chloramphenicol Erythromycin Gentamicin Kanamycin Nalidixic acid Norfloxacin Penicillin Tetracycline	5 ^1^ 5 ^1^ 100 ^1^ 2 ^1^ 10 ^1^ 4 ^1^ 0.1 ^1^ 100 ^1^ 4 ^1^	20 ^1^ 60 ^1^ 160 ^1^ 4 ^1^ 10 ^1^ 30 ^1^ 0.15 ^1^ 200 ^1^ 6 ^1^	- - - - n.a. - - - -	[8]
*Escherichia coli*	ATCC 47076	6-fold–7-fold	Chloramphenicol Florfenicol Ciprofloxacin Nalidixic acid Ampicillin Cefotaxime	8 ^1^ 8 ^1^ 0.06 ^1^ 8 ^1^ 4 ^1^ 0.06 ^1^	8–128 ^1^ 16–64 ^1^ 0.25 ^1^ 32–64 ^1^ 4–8 ^1^ 0.12–0.5 ^1^	- - - - - -	[9]
*Escherichia coli*	NCTC 12900 strain O157	Approx. 100-fold	Amoxicillin-clavulanic acid Amoxicillin Chloramphenicol Ciprofloxacin Clindamycin Colistin sulfate Erythromycin Fusidic acid Gentamicin Imipenem Rifampicin Tetracycline Trimethoprim Vancomycin	12 ^2^ 12 ^2^ 19 ^2^ 14 ^2^ 0 ^2^ 10 ^2^ 4 ^2^ 0 ^2^ 13 ^2^ 15 ^2^ 5 ^2^ 10 ^2^ 14 ^2^ 0 ^2^	0 ^2^ 0 ^2^ 0 ^2^ 14 ^2^ 0 ^2^ 104 ^2^ 0 ^2^ 13 ^2^ 10 ^2^ 5 ^2^ 4 ^2^ 0 ^2^ 0 ^2^	R R R n. a. n. a. n. a. n. a. n. a. n. a. R n. a. R R n. a.	[10]
*Escherichia coli* and *Salmonella* spp. (non-typhoidal)	12 pan-susceptible strains (6 per species)	24% ^4^	Tetracycline Ciprofloxacin Chloramphenicol Trimethoprim-Sulfamethoxazol Ampicillin Gentamicin	2.4 ^3,4^ 0.03 ^3,4^ 6.5 ^3,4^ 0.09 ^3,4^ 18.6 ^2,4^ 1.1 ^3,4^	23.3 ^3,4^ 0.11 ^3,4^ 13.7 ^3,4^ 0.14 ^3,4^ 12.0 ^2,4^ 1.3 ^3,4^	R (5) S I (6) S R (6) S	[11]
*Klebsiella oxytoca*	Biocide-sensitive strain from organic foods	3-fold	Ampicillin Cefotaxime Ciprofloxacin Imipenem Ceftazidime Tetracycline Trimethoprim-Sulfamethoxazol Sulfamethoxazol Nalidixic acid	No cross-tolerance ^1^ (all antibiotics)		n. a.	[7]
*Klebsiella* spp.	Biocide-sensitive strain from organic foods	36-fold	Ampicillin	-	64 ^1^	R	[7]
*Pantoea agglomerans*	Four biocide-sensitive strains from organic foods	20-fold–70-fold	Ampicillin Ceftazidime Cefotaxime	- - -	64 (4) ^1^ 32–64 (2) ^1^ 128 (1) ^1^	R R R	[7]
*Pantoea ananatis*	Biocide-sensitive strain from organic foods	25-fold	Ampicillin Cefotaxime Sulfamethoxazol	- - -	64 ^1^ 64 ^1^ 1024 ^1^	R R R	[7]
*Pantoea* spp.	Three biocide-sensitive strains from organic foods	100-fold–500-fold	Ampicillin Cefotaxime Sulfamethoxazol	- - -	64 (1) ^1^ 128 (1) ^1^ 1024 (1) ^1^	R R R	[7]
*Pseudomonas aeruginosa*	22 isolates from biofilm samples in dairy	≤2.2-fold	Ciprofloxacin	0.25–32 ^1^	3.5–55 ^1,5^	-	[12]
*Pseudomonas aeruginosa*	Strain NCIMB 10421	12-fold	Amikacin Ceftazidime Ciprofloxacin Gentamycin Imipenem Ticarcillin	3.5 ^3^ 2 ^3^ 0.125 ^3^ 2.5 ^3^ 2 ^3^ 0.875 ^3^	1.75 ^3^ 0.44 ^3^ 0.047 ^3^ 0.75 ^3^ 0.5 ^3^ 0.285 ^3^	n. a. n. a. n. a. n. a. n. a. n. a.	[13]
*Pseudomonas aeruginosa*	Strain NCIMB 10421	>12-fold	Ciprofloxacin Tobramycin Minocycline Aztreonam Polymyxin B Amikacin Gentamicin Vancomycin Imipenem	0.125 ^3^ 1.5 ^3^ >128 ^3^ 3 ^3^ 4 ^3^ 8 ^3^ 4 ^3^ >128 ^3^ 2 ^3^	32 ^3^ 1.0 ^3^ 16 ^3^ 3 ^3^ 2 ^3^ 6 ^3^ 6 ^3^ >128 ^3^ 2 ^3^	- - - - - - - - -	[14]
*Pseudomonas aeruginosa*	Isolate from river sediment	4-fold	Polymyxin B	0.2–0.4 ^1^	0.8–1.6 ^1^	-	[15]
*Salmonella Enteritidis*	Clinical isolate	Approx. 200-fold	Various antibiotics	No cross-resistance ^2^		n.a.	[10]
*Salmonella Hvittingfoss*	Strain S41	4-fold	Ampicillin Amoxicillin-clavulanic acid Piperacillin Cephalexin Cefpodoxime Ceftiofur Ceftriaxone Tetracycline Ciprofloxacin Chloramphenicol Cefoxitin Nalidixic acid	<2 ^6^ <2 ^6^ <4 ^6^ <4 ^6^ <0.25 ^6^ <1 ^6^ <0.25 ^6^ <1 ^6^ 0.06 ^6^ 4 ^6^ 8 ^6^ 4 ^6^	16 ^6^ 4 ^6^ 64 ^6^ 16 ^6^ 2 ^6^ >8 ^6^ 2 ^6^ 8 ^6^ 0.5 ^6^ 16 ^6^ >32 ^6^ 32 ^6^	I - I I I I R I I I - R	[16]
*Salmonella Typhimurium*	NCTC 74	Approx. 10-fold	Amoxicillin-clavulanic acid Amoxicillin Chloramphenicol Ciprofloxacin Clindamycin Colistin sulfate Erythromycin Fusidic acid Gentamicin Imipenem Rifampicin Tetracycline Trimethoprim Vancomycin	14 ^2^ 15 ^2^ 15 ^2^ 13 ^2^ 0 ^2^ 9 ^2^ 0 ^2^ 0 ^2^ 13 ^2^ 17 ^2^ 4 ^2^ 6 ^2^ 13 ^2^ 0 ^2^	14 ^2^ 14 ^2^ 15 ^2^ 15 ^2^ 0 ^2^ 9 ^2^ 0 ^2^ 0 ^2^ 11 ^2^ 16 ^2^ 4 ^2^ 9 ^2^ 13 ^2^ 0 ^2^	n. a. n. a. n. a. n. a. n. a. n. a. n. a. n. a. n. a. n. a. n. a. n. a. n. a. n. a.	[10]
*Salmonella Virchow*	Food isolate	Approx. 200-fold	Amoxicillin-clavulanic acid Amoxicillin Chloramphenicol Ciprofloxacin Clindamycin Colistin sulfate Erythromycin Fusidic acid Gentamicin Imipenem Rifampicin TetracyclineTrimethoprim Vancomycin	16 ^2^ 16 ^2^ 14 ^2^ 0 ^2^ 0 ^2^ 9 ^2^ 4 ^2^ 0 ^2^ 16 ^2^ 16 ^2^ 5 ^2^ 8 ^2^ 14 ^2^ 0 ^2^	0 ^2^ 1 ^2^ 2 ^2^ 0 ^2^ 0 ^2^ 11 ^2^ 4 ^2^ 0 ^2^ 15 ^2^ 12 ^2^ 5 ^2^ 8 ^2^ 0 ^2^ 0 ^2^	R R R n. a. n. a. n. a. n. a. n. a. n. a. R n. a. n. a. R n. a.	[10]

^1^ microdilution method (mg/L); ^2^ disc diffusion test (mm); ^3^ Etest (mg/L); ^4^ mean; ^5^ no conclusive cross-resistance; ^6^ NARMS plates; “-” = no information; R = resistant; I = intermediate susceptible; S = susceptible; n. a. = not applicable; () = number of strains, isolates or experiments.

**Table 3:** Gram-negative species with antibiotic resistance after various types of low level exposure (<MIC value) to chlorhexidine digluconate (CHG).

Species	Strain(s)	MIC Increase (CHG)	Antibiotic(s)	Pre-Value	Post-Value	Category	Reference
*Bacteroides fragilis*	ATCC 25285	-	Ampicillin Cefoxitin Cefoperazone Chloramphenicol Metronidazole Norfloxacin Tetracycline	46 ^1^ 7 ^1^ 52 ^1^ 2 ^1^ 0.6 ^1^ 0.6 ^1^ 0.6 ^1^	77 ^1^ 13 ^1^ 126 ^1^ 2 ^1^ 0.9 ^1^ 0.9 ^1^ 2 ^1^	- - - - - - -	[30]
*Burkholderia cepacia complex*	*B. lata* strain 383	-	Imipenem Meropenem Ciprofloxacin Ceftazidime Tobramycin	24 ^2^ 40.7 ^2^ 30 ^2^ 40.3 ^2^ 7.3 ^2^	15–21 (2) ^2^ 33 (1) ^2^ 11–20 (2) ^2^ 30–33 (2) ^2^ -	- - - - -	[6]
*Chrysobacterium* spp.	2 biocide-sensitive strains from organic foods	5-fold–6-fold	Ampicillin Cefotaxime Ceftazidime Sulfamethoxazol Tetracycline	- - - - -	64 (1) ^2^ 128 (2) ^2^ 64 (2) ^2^ 1024 (1) ^2^ 16 (1) ^2^	R R R R R	[31]
*Enterobacter cloacae*	2 biocide-sensitive strains from organic foods	10-fold–16-fold	Cefotaxime Ceftazidime Imipenem Sulfamethoxazol Tetracycline	- - - - -	64 (1) ^2^ 64 (2) ^2^ 16 (2) ^2^ 1024 (2) ^2^ 32 (1) ^2^	R R R R R	[31]
*Enterobacter ludwigii*	2 biocide-sensitive strains from organic foods	6-fold–8-fold	Ceftazidime Imipenem Sulfamethoxazol	- - -	64 (2) ^2^ 16 (2) ^2^ 1024 (2) ^2^	R R R	[31]
*Enterobacter* spp.	6 biocide-sensitive strains from organic foods	4-fold–10-fold	Cefotaxime Ceftazidime Imipenem Sulfamethoxazol	- - - - -	64 (1) ^2^ 128 (1) ^2^ 64 (3) ^2^ 16 (3) ^2^ 1024 (2) ^2^	R R R R R	[31]
*Escherichia coli*	NCIMB 8545	≤6-fold	Tobramycin	-	- ^2^	R ^3^	[32]
*Escherichia coli*	NCTC 12900 strain O157	Approx. 50-fold	Various antibiotics	No cross-resistance ^4^		n.a.	[10]
*Klebsiella oxytoca*	2 biocide-sensitive strains from organic foods	2-fold–8-fold	Various antibiotics	No cross-resistance ^2^		n.a.	[31]
*Klebsiella pneumoniae*	6 clinical strains with a variety of antibiotic resistance markers	4-fold–16-fold	Azithromycin Cefepime Colistin Teicoplanin	8–64 (6) 0.06–0.125 (1) ≥64 (5) 2–4 (6) >64 (6)	8–64 (6) ^2^ 0.06–0.5 (2) ^2^ ≥64 (4) ^2^ >64 (5) ^2^ >64 (6) ^2^	n.a. n.a. n.a. R n.a.	[33]
*Klebsiella* spp.	Biocide-sensitive strain from organic foods	2-fold	Ceftazidime Imipenem	- -	64 ^2^ 16 ^2^	R R	[31]
*Pantoea agglomerans*	5 biocide-sensitive strains from organic foods	5-fold–10-fold	Cefotaxime Ceftazidime Imipenem Sulfamethoxazol Tetracycline	- - - - -	64–128 (3) ^2^ 64 (3) ^2^ 16 (1) ^2^ 1024 (2) ^2^ 16–32 (2) ^2^	R R R R R	[31]
*Pantoea ananatis*	2 biocide-sensitive strains from organic foods	10-fold–50-fold	Cefotaxime Ceftazidime Imipenem Sulfamethoxazol Tetracycline	- - - - -	64–128 (2) ^2^ 64 (1) ^2^ 16 (1) ^2^ 1024 (1) ^2^ 16 (1) ^2^	R R R R R	[31]
*Pantoea* spp.	3 biocide-sensitive strains from organic foods	5-fold–16-fold	Ampicillin Cefotaxime Ceftazidime Imipenem Sulfamethoxazol Tetracycline	- - - - - -	32 (1) ^2^ 128 (1) ^2^ 64 (1) ^2^ 16 (1) ^2^ 1024 (1) ^2^ 16–32 (2) ^2^	R R R R R R	[31]
*Salmonella Virchow*	Food isolate	Approx. 10-fold	Various antibiotics	No cross-resistance ^4^		n.a.	[10]
*Salmonella* spp.	3 biocide-sensitive strains from organic foods	5-fold–10-fold	Cefotaxime Imipenem Nalidixic acid Sulfamethoxazol Tetracycline	- - - -	128 (2) ^2^ 16 (2) ^2^ 64 (2) ^2^ 1024 (1) ^2^ 32 (1) ^2^	R R R R R	[31]
*Salmonella* spp.	6 strains with higher MICs to biocidal products	50-fold–200-fold (2 strains)	Tetracycline Chloramphenicol Nalidixic acid	<1 ^4^ 4 ^4^ 4 ^4^	>16 (1) ^5^ 8 (1) ^5^ 16 (1) ^5^	R I I	[16]

^1^ spiral gradient endpoint method (mg/L); ^2^ microdilution method (mg/L); ^3^ unstable; ^4^ disc diffusion test (mm); ^5^ NARMS plates (mg/L); - no information; R = resistant; I = intermediate susceptible; S = susceptible; () number of strains or isolates.

**Table 4:** Gram-negative species with antibiotic resistance after various types of low level exposure (<MIC value) to triclosan (TRI).

Species	Strain(s)	MIC Increase (TRI)	Antibiotic(s)	Pre-Value	Post-Value	Category	Reference
*Actinomyces naeslundii*	Strain WVU627	4.9-fold	Metronidazole Tetracycline	125 ^1^ 5.2 ^1^	125 ^1^ 7.8 ^1^	- -	[50]
*Enterobacter* spp.	5 biocide-sensitive strains from organic foods	2-fold–15-fold	Ampicillin Cefotaxime Ceftazidime Sulfamethoxazol	- - - -	64 (2) ^1^ 128 (1) ^1^ 64 (2) ^1^ 1024 (2) ^1^	R R R R	[51]
*Escherichia coli*	ATCC 8729	391-fold	Metronidazole Tetracycline	250 ^1^ 15.6 ^1^	125 ^1^ 10.4 ^1^	- -	[50]
*Escherichia coli*	NCTC 12900 strain O157	16-fold (P1)8192-fold (P2)	Amoxicillin-clavulanic acid Amoxicillin Chloramphenicol Ciprofloxacin Clindamycin Colistin sulfate Erythromycin Fusidic acid Gentamicin Imipenem Rifampicin Tetracycline Trimethoprim Vancomycin	11 ^2^ 13 ^2^ 13 ^2^ 14 ^2^ 0 ^2^ 9 ^2^ 7 ^2^ 0 ^2^ 12 ^2^ 15 ^2^ 5 ^2^ 17 ^2^ 13 ^2^ 0 ^2^	0 ^2^ 0 ^2^ 5 ^2^ 14 ^2^ 0 ^2^ 10 ^2^ 0 ^2^ 0 ^2^ 12 ^2^ 11 ^2^ 5 ^2^ 14 ^2^ 0 ^2^ 0 ^2^	R R R n. a. n. a. n. a. R n. a. n. a. R n. a. R R n. a.	[10]
*Escherichia coli*	ATCC 27325	4096-fold	Amoxicillin Amoxicillin-clavulanic acid Chloramphenicol Ciprofloxacin Clindamycin Colistin sulfate Fusidic acid Gentamicin Rifampicin Tetracycline Trimethoprim Vancomycin	8 ^1^ 8 ^1^ 16 ^1^ 4 ^1^ >256 ^1^ 16 ^1^ >256 ^1^ 8 ^1^ 256 ^1^ 32 ^1^ 32 ^1^ >256 ^1^	8 ^1^ 8 ^1^ 256 ^1^ 4 ^1^ >256 ^1^ 16 ^1^ >256 ^1^ 8 ^1^ 256 ^1^ 32 ^1^ 32 ^1^ >256 ^1^	n. a. n. a. R n. a. n. a. n. a. n. a. n. a. n. a. n. a. n. a. n. a.	[52]
*Escherichia coli*	Strain O55:H7	2048-fold	Amoxicillin Amoxicillin-clavulanic acid Chloramphenicol Ciprofloxacin Clindamycin Colistin sulfate Fusidic acid Gentamicin Rifampicin Tetracycline Trimethoprim Vancomycin	8 ^1^ 16 ^1^ 16 ^1^ 2 ^1^ >256 ^1^ 16 ^1^ >256 ^1^ 8 ^1^ >256 ^1^ 32 ^1^ 32 ^1^ 0 ^1^	8 ^1^ 8 ^1^ 8 ^1^ 2 ^1^ >256 ^1^ 16 ^1^ >256 ^1^ 16 ^1^ >256 ^1^ 32 ^1^ 256 ^1^ 0 ^1^	n. a. n. a. n. a. n. a. n. a. n. a. n. a. n. a. n. a. n. a. R n. a.	[52]
*Escherichia coli*	NCTC 12900	8192-fold	Amoxicillin Amoxicillin-clavulanic acid Chloramphenicol Ciprofloxacin Clindamycin Colistin sulfate Fusidic acid Gentamicin Rifampicin Tetracycline Trimethoprim Vancomycin	32 ^1^ 4 ^1^ 32 ^1^ 2 ^1^ >256 ^1^ 8 ^1^ >256 ^1^ 16 ^1^ >256 ^1^ 32 ^1^ 64 ^1^ 0 ^1^	>256 ^1^ 256 ^1^ 256 ^1^ 2 ^1^ >256 ^1^ 16 ^1^ >256 ^1^ 16 ^1^ >256 ^1^ >256 ^1^ >256 ^1^ 0 ^1^	R R R n. a. n. a. n. a. n. a. n. a. n. a. R R n. a.	[52]
*Fusobacterium nucleatum*	ATCC 10953	None	Metronidazole Tetracycline	250 ^1^ 3.9 ^1^	500 ^1^ 2.9 ^1^	- -	[50]
*Neisseria subflava*	Strain A1078	None	Metronidazole Tetracycline	62.5 ^1^ 3.9 ^1^	52.1 ^1^ 6.8 ^1^	- -	[50]
*Pantoea agglomerans*	Biocide-sensitive strain from organic foods	150-fold	Ampicillin Ceftazidime Sulfamethoxazol	- - -	64 ^1^ 64 ^1^ 1024 ^1^	R R R	[51]
*Pantoea ananatis*	2 biocide-sensitive strains from organic foods	5-fold– 200-fold	Sulfamethoxazol Trimethoprim-sulfamethoxazol Ampicillin Cefotaxime	- - - -	1024 (2) ^1^ 8/152 (2) ^1^ 32 (1) ^1^ 64 (1) ^1^	R R R R	[51]
*Pantoea* spp.	2 biocide-sensitive strains from organic foods	2-fold–3-fold	Sulfamethoxazol Ceftazidime Cefotaxime	- - -	1024 (1) ^1^ 64 (1) ^1^ 128 (1) ^1^	R R R	[51]
*Porphyromonas gingivalis*	Strain W50	None	Metronidazole Tetracycline	31.3 ^1^ 3.0 ^1^	62.5 ^1^ 1.0 ^1^	- -	[50]
*Prevotella nigrescens*	Strain T588	2-fold	Metronidazole Tetracycline	62.5 ^1^ 1.0 ^1^	62.5 ^1^ 1.0 ^1^	- -	[50]
*Salmonella* spp.	3 biocide-sensitive strains from organic foods	2-fold– 200-fold	Trimethoprim-sulfamethoxazol Cefotaxime Nalidixic acid Ampicillin Sulfamethoxazol Imipenem	- - - - - -	8/152 (2) ^1^ 64/128 (2) ^1^ 64 (2) ^1^ 64 (1) ^1^ 1024 (1) ^1^ 32 (1) ^1^	R R R R R R	[51]
*Salmonella* spp.	6 strains with higher MICs to biocidal products	500-fold– 10.000-fold (3)	Piperacillin Ceftiofur Amikacin Gentamicin Kanamycin Chloramphenicol Cefoxitin Nalidixic acid Sulfisoxazole	<4 ^3^ 2 ^3^ 4 ^3^ <1 ^3^ <8 ^3^ 4 ^3^ 16 ^3^ 8 ^3^ 32 ^3^	16 ^3^ >8 ^3^ 16 ^3^ 4 ^3^ 32 ^3^ 16 ^3^ 32 ^3^ 32 ^3^ >256 ^3^	I R I I I I R R I	[16]
*Veillonella dispar*	ATCC 17745	None	Metronidazole Tetracycline	78.1 ^1^ 31.3 ^1^	31.3 ^1^ 27.4 ^1^	- -	[50]

^1^ microdilution method (mg/L); ^2^ disc diffusion test (mm); ^3^ NARMS plates (mg/L); “-” = no information; R = resistant; I = intermediate susceptible; S = susceptible; n. a. = not applicable; () = number of strains, isolates or experiments; (P1) = passage 1; (P2) = passage 2.

**Table 5:** Gram-negative species with antibiotic tolerance or resistance after low level exposure (< MIC value) to didecyldimethylammonium chloride (DDAC).

Species	Strain(s)	Type of DDAC Exposure	Antibiotic(s)	Reference
*Escherichia coli*	54 strains from pig faeces or pork meat	7 d at various concentrations.	32 strains became multiresistant, most of them with a new resistance ^1^ to chloramphenicol, ampicillin, cefotaxime, ceftazidime and ciprofloxacin	[59]
*Salmonella enterica*	54 strains from pig faeces or pork meat	7 d at various concentrations	7 strains acquired a new resistance ^1^, mainly to chloramphenicol (3 strains)	[59]

^1^ microdilution method (mg/L).
